# Liquid Biopsy: An Evolving Paradigm for Non-invasive Disease Diagnosis and Monitoring in Medicine

**DOI:** 10.7759/cureus.50176

**Published:** 2023-12-08

**Authors:** Kanishk K Adhit, Anil Wanjari, Sharanya Menon, Siddhaarth K

**Affiliations:** 1 Medicine, Jawaharlal Nehru Medical College, Datta Meghe Institute of Higher Education and Research, Wardha, IND; 2 Pathology, Jawaharlal Nehru Medical College, Datta Meghe Institute of Higher Education and Research, Wardha, IND

**Keywords:** cell-free dna, ctdna, circulating exosomes, circulating tumor cells (ctcs), precision medicine, liquid biopsy

## Abstract

Liquid biopsy stands as an innovative instrument in the realm of precision medicine, enabling non-invasive disease diagnosis and the early detection of cancer. Liquid biopsy helps in the extraction of circulating tumor DNA (ctDNA), circulating tumor cells (CTCs), and cell-free DNA (cfDNA) from blood samples and other body fluids, thereby facilitating disease diagnosis and prediction of high-risk patients. Various techniques such as advanced sequencing methods and biomarker-based cell capture have led to the isolation and study of the different biomarkers such as ctDNA, cfDNA, and CTCs. These biopsies also have immense potential in the early detection and diagnosis of various diseases across all medical specialties, prediction and screening of high-risk cases, and detection of different immune response patterns in response to infectious diseases, and also help in predicting treatment outcomes. Although liquid biopsy has the potential to disrupt the field of medical diagnosis, it is met by various challenges such as limited tumor-derived components, less specificity, and inadequate advancement in methods to isolate biomarkers. Despite all these challenges, liquid biopsies provide the potential to become a minimally invasive method of diagnosis that would facilitate real-time monitoring of patients, which differentiates them from traditional tissue biopsies. This article aims to provide a complete overview of the current technologies, different biomarkers, and body fluids that can be used in liquid biopsy and its clinical applications and the potential impact that liquid biopsy holds in the field of precision medicine, facilitating early diagnosis and prompt management of various diseases and cancers.

## Introduction and background

**Figure 1:**
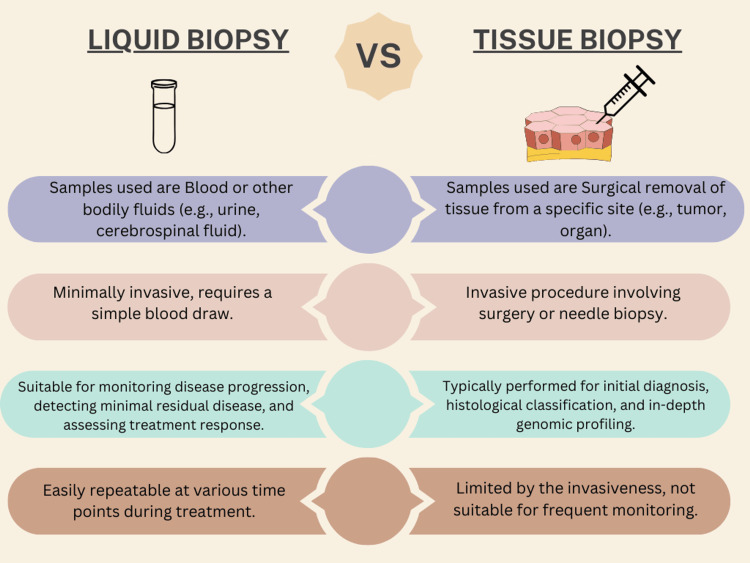
Comparison between liquid biopsy and traditional tissue biopsy Figure Credit: Author Kanishk K Adhit

Liquid biopsy is a new emerging precision medicine technique that analyses biological fluids, including blood, to get additional information about a patient's health. It is a non-invasive technique for identifying and analyzing biomarkers such as circulating tumor cells (CTCs), cell-free DNA (cfDNA), and exosomes. It allows for real-time monitoring of a patient's cancer mutations and genomic profile, which facilitates more customized and adaptable treatment options. It can be applied to many different facets of cancer therapy, including early cancer detection, treatment selection, resistance and response monitoring, and detection of mildly recurring disease or recurrence. Liquid biopsies are quicker, safer, and more practical than conventional tissue biopsies, and they also allow for serial samples to monitor tumor evolution over time [1]. The comparison between traditional biopsy and liquid biopsy is shown in Figure 1.

Precision medicine is the personalization of healthcare with medical decisions, practices, treatments, and goods suited to a specific patient based on their genetic makeup, environment, lifestyle, and other factors. Instead of taking a one-size-fits-all approach, the objective is to give the right treatment to the right patient at the right time. Technologies and diagnostic procedures make this possible. It covers disease prevention, diagnosis, treatment, and aftercare. Molecular diagnostics, medical imaging, and big data analytics are examples of tools that are used to demonstrate precision medicine. Advances in genomes, medical imaging, health information technology, diagnostics, and tailored medicines have made precision medicine more prevalent [2].

The clinical uses of liquid biopsy in medicine extend to many specialties such as oncology, cardiology, prenatal testing, infectious diseases, and neurology [3]. Serial liquid biopsies, as opposed to traditional tissue biopsies, can be used to characterize and monitor the progression of a disease and provide more individualized data to examine heterogeneity in presentation, select the best therapy, assess therapeutic response, and find potential resistance mechanisms or relapse indicators. Numerous developments, including advancements in lab-on-a-chip technology, nanotechnology, microfluidics, and next-generation sequencing, have improved analytical sensitivity and effectiveness for profiling circulating biomarkers released into biofluids or shed from tumors [4]. While liquid biopsy applications span diverse disease areas, oncology has been at the forefront of translating these techniques into clinical care. The treatment of cancer starts with obtaining an effective diagnosis; for diagnosis, the most commonly used modalities in current times are invasive biopsy procedures, which are required to collect the samples required for molecular diagnostic testing.

Through liquid biopsy, molecular diagnostic testing can be done using a normal blood test itself, which is less invasive than traditional biopsies. Liquid biopsy is reshaping all aspects of cancer management including early screening and detection, molecular characterization of tumors, guiding targeted therapy selection, assessing the efficacy of treatments, monitoring minimal residual disease, tracking mutations associated with drug resistance, and enabling surveillance for recurrence [5]. In 2010, Catherine Alix-Panabières and Klaus Pantel first used the term liquid biopsy in a paper examining the difficulties and possibilities of CTCs in cancer. Although the phrase liquid biopsy was originally solely used to refer to CTCs, it was immediately extended to include another circulating biomarker, circulating tumor DNA (ctDNA) [6]. Research in the area of liquid biopsy rapidly increased once CTCs and ctDNA were recognized as the two essential components. In the majority of solid tumor types, including breast, prostate, lung, and colorectal, CTCs and ctDNA have been identified as predictive indicators. Studies are still ongoing with the goal of proving the clinical utility of CTCs and ctDNA in areas such as early cancer identification, enhanced cancer staging, relapse detection, and monitoring of response to therapy in real time.

As a result, numerous technologies are developed to be used in the identification, enrichment, and description of ctDNA and CTCs, as well as numerous assays that can be used on the targets. This review synthesizes recent advances in liquid biopsy approaches, evaluates the strength of evidence and clinical utility across different applications, and offers an outlook on the future as liquid biopsy gains acceptance as a mainstream paradigm for non-invasive diagnosis and dynamic disease monitoring. 

## Review

Methodology

**Figure 2:**
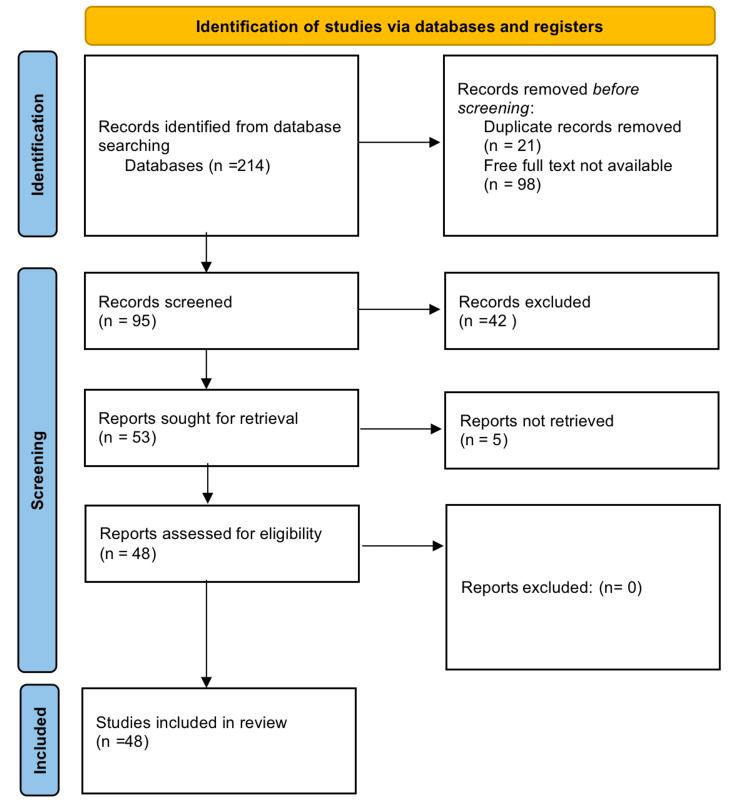
PRISMA flow diagram of search strategy PRISMA: Preferred Reporting Items for Systematic Reviews and Meta-Analyses

For gathering all the required papers for this review article, a thorough literature search technique was needed in the methodology section of this narrative review. Google Scholar and PubMed were the two electronic databases accessed. The following keywords were used: "liquid biopsy," "precision medicine," ("liquid biopsy" [MeSH Terms]) AND (("precision medicine" [MeSH Terms]). The inclusion of both original research articles and review papers was taken into consideration. To ensure that the inclusion of studies satisfies the established criteria, the selection procedure comprised of evaluating article titles, abstracts, and full texts. We additionally searched for key references from bibliographies of the relevant studies. A Preferred Reporting Items for Systematic Reviews and Meta-Analyses (PRISMA) flow diagram is used to demonstrate the number of studies included (Figure 2).

What is liquid biopsy?

Liquid biopsy is used to analyze the components of tumors that are released into the blood and other biofluids from the main tumor and metastatic sites, such as CTCs, ctDNA, cfRNA, and exosomes. These elements can be isolated from typical blood samples and then analyzed to offer essential real-time information about tumor molecular profiling, treatment response monitoring, identification of resistance mechanisms, and evaluation of minimal residual disease. Liquid biopsies provide serial, non-invasive sampling that can capture tumor heterogeneity and follow disease evolution in response to therapy or throughout progression, as opposed to typical surgical tumor tissue biopsies that only offer a picture of one lesion at a single time point [7]. The effective analysis of circulating biomarkers released into the bloodstream by tumors has been made possible by developments in sensitive molecular technologies like next-generation sequencing and microfluidic platforms. These developments also hold the promise of revolutionizing personalized medicine approaches for screening, diagnosis, treatment selection, and surveillance across most solid malignancies and some hematologic cancers.

Liquid biopsy techniques and biomarkers

CTCs

CTCs represent a powerful biomarker that can be isolated and analyzed through liquid biopsy approaches. First discovered in 1869 by Thomas Ashworth, CTCs are cancer cells shed into the bloodstream from primary or metastatic tumor sites [8]. These rare cells can be identified based on immunoaffinity capture techniques and the expression of certain epithelial markers [9]. A major challenge is that CTCs exhibit high heterogeneity, enabling evasion of immune surveillance and resistance to therapies, leading to distal metastases and recurrence [10]. Moreover, CTCs have a short half-life of just 1-2.4 hours in circulation, existing at extremely low concentrations in peripheral blood [11]. Analysis of CTCs through liquid biopsy has been employed for decades across numerous cancer types, demonstrating utility for early diagnosis, prognostic risk stratification, dynamic disease monitoring, and guiding personalized therapeutic decisions [12]. Moving forward, advances in isolation and analytic techniques will continue to unlock the promise of CTCs as circulating biomarkers for precision oncology applications.

cfDNA Analysis

The discovery of cfDNA fragments circulating in blood was found by Mandel in 1948; he coined the term cfDNA [13]. A major breakthrough came in 1994 when Thierry et al. utilized analysis of cfDNA to detect specific genetic and mutational tumor changes, establishing the concept of ctDNA [14]. We now understand that ctDNA represents a subset of cfDNA that originates from apoptotic and necrotic primary tumor cells, metastatic lesions, or CTCs released into the circulation [15]. Research has shown that ctDNA levels can be impacted by multiple factors including tumor burden, stage, clearance rates, inflammation, and tissue damage or cell turnover, allowing ctDNA analysis to provide dynamic monitoring of tumorigenesis and progression [16]. Additionally, ctDNA carries a wide spectrum of tumor-specific genetic and epigenetic alterations, including mutations, copy number variations, and aberrant methylation patterns [17]. Therefore, ctDNA liquid biopsy analysis enables non-invasive detection of inter- and intra-tumor heterogeneity over time, which can guide personalized therapeutic approaches for cancer screening and monitoring.

Other Biomarkers

In addition to CTCs and cfDNA, extracellular vesicles (EVs), microRNAs (miRNAs), and proteins represent emerging classes of informative biomarkers for liquid biopsy analysis. EVs such as exosomes are lipid bilayer vesicles containing proteins, RNAs, and DNAs released by both normal and cancerous cells that can reflect physiology and pathologic states [18]. Certain miRNA patterns in body fluids have been associated with cancer diagnosis, prognosis, and treatment response monitoring [19]. Analysis of circulating proteins including specific antigens, metabolites, and tumor-educated blood platelets may also enable minimally invasive characterization of the tumor microenvironment [20]. Integrated analysis of CTCs, cfDNA, EVs, miRNAs, proteins, and other molecules in biofluids provides a more comprehensive real-time molecular profile of disease heterogeneity than any single analyte alone. However, rigorous assessment of clinical validity and utility is still needed prior to the routine adoption of these emerging approaches.

Utility of various biofluids for liquid biopsy analysis

Blood

Blood has been the predominant biofluid utilized for liquid biopsy analysis, enabling minimally invasive detection of CTCs, cfDNA, exosomes, and other analytes shed from primary and metastatic tumor sites. Blood-based liquid biopsies have shown utility across cancer types including lung, gastrointestinal, breast, and hematological malignancies [21].

Urine 

Urine contains tumor-derived cfDNA, exosomes, metabolites, and other constituents that originate from circulation and reflect pathologies in the urinary tract as well as cancers and disorders across the body. Urine liquid biopsies thus enable completely non-invasive detection and monitoring of diseases like prostate, bladder, and kidney cancer [22].

Saliva

Saliva contains diverse analytes including proteins, nucleic acids, and metabolites that strongly correlate with serum levels. This makes saliva well-suited for assessing oral diseases, oral cancer, pancreatic cancer, gastric cancer, various systemic conditions like diabetes mellitus, acute myocardial infarction, Parkinson's disease, and autoimmune disorders such as Sjogren's syndrome, systemic lupus erythematosus (SLE), rheumatoid arthritis, and coeliac disease. The non-invasive method of sample collection makes saliva an attractive biofluid for liquid biopsy development [23,24].

Breast Milk

Breast milk provides a window into the local environment of the breast through exfoliated epithelial cells and vesicles containing proteins and nucleic acids reflective of physiological status. Analyzing these components may enable the screening of asymptomatic women by identifying circulating biomarkers shed from occult breast tumors such as ductal carcinoma in situ (DCIS), lobular carcinoma in situ (LCIS), invasive lobular carcinomas (ILCs), and metastatic breast cancers with an unknown primary site [25].

Synovial Fluid

Synovial fluid obtained from certain diseases causing inflammation of joints such as osteoarthritis and rheumatoid arthritis contains exosomes, non-coding RNAs, and proteins participating in cell signaling and inflammation. Biomarker identification in synovial fluid can allow the detection of joint degradation and diagnosis of diseases such as rheumatoid arthritis, osteoarthritis, gout, and septic arthritis [26].

In summary, while blood remains a versatile liquid biopsy biofluid, analyzing additional bodily fluids through minimally invasive approaches provides complementary opportunities for diagnosis, prognosis, and disease monitoring across oncology and other disciplines.

Liquid biopsy applications in oncology

Liquid Biopsy as a Point-of-Care Diagnostic

A point-of-care diagnostic is a diagnostic tool that can be used at the site of the patient where the patient is located. The advantages of point-of-care diagnostics are that they provide faster results and enable rapid testing, often in less than 30 minutes while the patient is still onsite, thereby allowing healthcare workers to make rapid clinical decisions and improve their efficiency. It is also an easy-to-use diagnostic test that does not require any highly trained personnel or sample collection and processing techniques and thereby can be used without the requirement of elaborate lab infrastructure. It is also a portable device that is compact and movable, thereby enabling bedside testing. Point-of-care diagnostic technologies are advancing liquid biopsy approaches by enabling rapid analysis of disease biomarkers from biofluids at a patient’s bedside. For example, microfluidic lab-on-a-chip devices can integrate processing and detection of circulating biomarkers with high throughput and accuracy. Other technologies like portable surface-enhanced Raman spectroscopy systems, plasmonic sensors, and paper-based microfluidic sensors provide the means to quantify cancer protein markers or tumor DNA from finger-prick blood samples within minutes. Such decentralized testing can facilitate routine monitoring and screening without needing complex instruments. However, further advances around multiplexing biomarker panels and ease of use are still required to translate these platforms effectively to widespread point-of-care use [27].

Early Cancer Detection and Screening

Liquid biopsy approaches have shown promising clinical utility for early detection of cancer and the screening of asymptomatic populations. Plasma screening tests analyzing circulating proteins, tumor-derived extracellular vesicles, and ctDNA mutations have achieved the detection of occult malignancies across cancer types at localized stages when curative treatment is still possible. Multi-analyte blood tests assess tumor-educated platelets containing RNA biomarkers as well as circulating metabolites, antigens, and mutant ctDNA to detect signals of early cancers with high accuracy. CancerSEEK evaluated levels of circulating proteins and mutations in ctDNA to detect eight common cancer types with a median sensitivity of 70%. CancerSEEK is a liquid biopsy assay that can be used for the detection of multiple cancer types; it is a non-invasive blood assay that can detect common carcinomatous conditions such as lung cancer, ovarian cancer, colorectal cancer, breast cancer, carcinoma of the esophagus and pancreatic cancer. It works by analyzing blood samples for the presence of ctDNA and biomarkers such as carcinoembryogenic antigen (CEA), cancer antigen 125 (CA-125), and others. By detecting these biomarkers and ctDNA signatures, it can identify the cell of the organ of the cancer and also can pinpoint and locate the tumor. In a study, CancerSEEK was tested on 1005 patients with cancer; it detected over 70 percent of the cancers with varying sensitivities depending on the cancer subtype. This test has great potential to be used as an early diagnostic tool to diagnose cancer that is faster than traditional biopsy techniques [28]. These non-invasive screening approaches may enable detection at curable stages and reduce cancer mortality. However, large collaborative trials are still needed to firmly establish its utility in clinical scenarios and to justify broader clinical implementation.

Detection of Minimal Residual Disease

MRD, or minimal residual disease, is the presence of residual tumor cells or sparse malignant cells that cannot be detected using traditional methods or regular radiological tests. There is a chance that a liquid biopsy will find MRD. Through CTC, ctDNA, or tumor-specific miRNA, it examines tumor-specific changes in the blood. Liquid biopsy can also find changes in DNA methylation. As a standalone predictive marker for the high risk of relapse in some forms of cancer, abnormal methylation patterns in cancer cells have demonstrated encouraging outcomes. However, the methylation pattern-based MRD identification lacks sufficient specificity for everyday clinical application [29].

Monitoring Treatment Response and Disease Progression

Liquid biopsies enable real-time monitoring of molecular profiles during therapy to assess treatment response and early progression. Tracking genetic mutations in serial ctDNA measurements allows the detection of emerging resistance to targeted therapies, enabling rapid adaptation of treatment plans [30]. In metastatic colorectal cancer, quantitative ctDNA dynamics showed a correlation to radiologic progression-free survival and overall survival [31]. For metastatic breast cancer, changes in ctDNA levels predicted prognosis and outcomes with different therapies [32]. Serial ctDNA assessment also enables early evaluation of chemotherapy, radiation, or surgical efficacy by monitoring molecular residual disease [33]. Overall, longitudinal liquid biopsies allow dynamic tracking of tumor evolution during therapy. This supports timely change in ineffective treatments or interventions upon early progression detection, with the goal of improving clinical outcomes.

Comparison Between Liquid Biopsy and Tissue Biopsy for Cancer Detection

Liquid biopsy analyzes tumor DNA in blood or other body fluids and thereby offers certain advantages over traditionally used tissue biopsy for diagnosis and monitoring of cancer. Liquid biopsy is a minimally invasive diagnostic tool when compared with tissue biopsy. It can be repeatedly used to track the evolution of tumors, and it also helps to better capture intra-tumor heterogeneity compared to that from a single tissue biopsy. However, liquid biopsy also has its own set of limitations, thereby leading to reduced sensitivity, specificity, and lack of standardization compared to currently established tissue biopsy. Tissue biopsy remains the gold standard in cancer diagnosis with better accuracy and reliability. However, the limitations of traditional tissue biopsy are that it is more invasive, provides limited snapshots of tumor biology, and cannot be serially performed to look for tumor evolution [34]. 

Liquid biopsy beyond oncology

Cardiovascular Diseases and Liquid Biopsy Markers

While oncology has been the primary focus, liquid biopsy approaches are gaining traction in cardiovascular medicine to enable minimally invasive molecular profiling and dynamic monitoring. Analysis of cfDNA and circulating miRNAs in plasma have shown promise as biomarkers for diverse cardiac indications including myocardial infarction, heart failure, ischemia, hypertension, transplant rejection, and stroke [35,36]. Specific miRNA signatures released during myocardial necrosis provided accurate early detection of myocardial infarction while circulating miRNAs have also been associated with the severity of heart failure and its outcomes [37]. Beyond diagnosis, serial assessment of cfDNA and miRNAs may allow monitoring of disease progression, treatment response, and prognostic risk stratification. Ongoing multi-center trials continue to assess the clinical validity and utility of circulating biomarkers for precision cardiovascular medicine applications from screening to guided therapy. Standardization of high-sensitivity lab and analytic techniques tailored to cardiac liquid biopsy will be key to enabling broader clinical translation and routine adoption of these approaches beyond oncology.

Liquid Biopsy Applications in Infectious Diseases

Liquid biopsy through analysis of microbial cfDNA (mcfDNA) in blood shows promise for improving diagnosis and management of bloodstream infections. mcfDNA sequencing enables the identification of causative pathogens in sepsis faster than standard blood culture, with higher detection rates of approximately 20-30% [38]. Case reports have studied the use of mcfDNA tests, which are used in promptly diagnosing infections caused by fastidious organisms, such as *Propionibacterium*, *Chlamydia*, and *methicillin-resistant Staphylococcus aureus (MRSA)*, that evade detection by conventional microbiology [39]. Tuberculosis (TB) serves as a pertinent example of an infectious ailment that could greatly benefit from cfDNA sequencing testing. The clinical identification of TB is hindered due to its prolonged latency period and vague initial symptoms. Diagnosis often takes more time as we are solely dependent on acid-fast bacillus (AFB) culture, and other traditional cultural methods are frequently required to culture the microorganisms in cases of invasive infections.

Researchers have developed targeted cfDNA assays, to detect *Mycobacterium tuberculosis* infection. These assays, conducted on blood and urine specimens, showcase the potential of cfDNA as a valuable biomarker for early TB detection and treatment monitoring. Recent evaluations of cfDNA sequencing tests in TB-infected patients have yielded positive results [40]. Similarly, successful applications have been observed in HIV patients suffering from infections of *Mycobacterium avium* and *Mycobacterium tuberculosis *[41]. These instances emphasize the potential of this novel approach as a less invasive and promising diagnostic and monitoring tool for TB. The widespread utilization of immune-suppressive treatment strategies and the emergence of antifungal-resistant microorganisms have given rise to cases of invasive fungal infections, which persist as a significant source of illness and death in individuals with compromised immune systems [42]. Given the extensive variety of causative fungal agents, there exists a pressing demand for swift and non-invasive methods of pinpointing the exact species involved in these invasive infections. This is essential for tailoring precise antifungal treatments. In 2018, a study found the application of cfDNA sequencing among patients with confirmed invasive fungal diseases. Remarkably, the study managed to identify the same fungus found in biopsy samples. This groundbreaking research highlighted the potential of identifying pathogenic cfDNA from deeply entrenched infections attributed to challenging-to-cultivate molds like *Aspergillus, Rhizomucor, *and* Scedosporium* species [43]. These identifications were made directly from sequenced plasma samples, potentially facilitating swifter diagnoses and negating the necessity for invasive biopsy procedures.

Neurological Disorders and Potential Biomarkers

**Figure 3:**
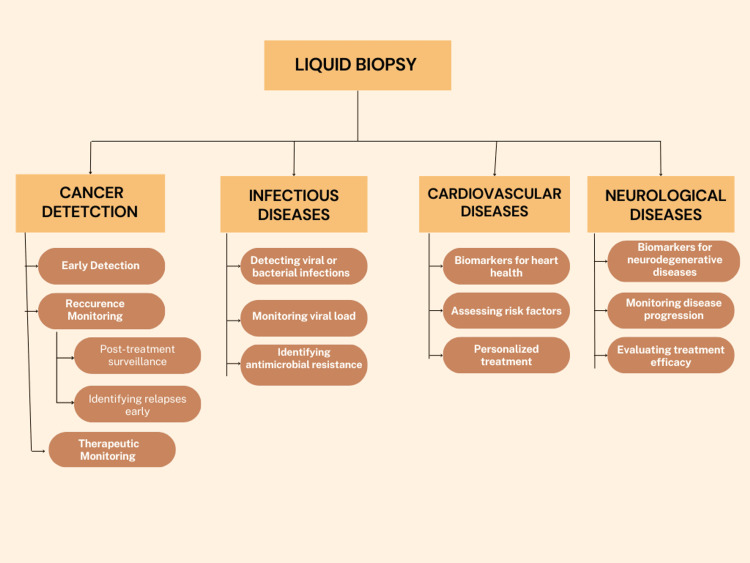
Applications of liquid biopsy Figure Credit: Author Kanishk K Adhit

Neurological diseases such as Parkinson's disease, multiple sclerosis, and Alzheimer's disease lack easily accessible diagnostic markers for early detection and disease surveillance. Liquid biopsy through analysis of extracellular vesicles that are derived from the brain and cfDNA in blood represents a non-invasive type of approach to detect specific biomarkers associated with various neurodegenerative disorders [44]. Specific miRNA signatures in plasma exosomes and cerebrospinal fluid extracellular vesicles have been associated with Parkinson’s disease and Alzheimer’s disease [45]. With further validation, a liquid biopsy of brain-derived biomarkers from biofluids has the potential to enable earlier diagnosis, improved prognostication, and tracking of treatment response for major neurological disorders using a simple blood test. A diagrammatic representation of the application of liquid biopsy is shown in Figure 3.

Challenges and limitations

One of the key limitations of liquid biopsy is low sensitivity; liquid biopsies when compared to traditional tissue biopsies have lower sensitivity in the detection of genetic mutations and cancer biomarkers. The main reason for this is the presence of low levels of ctDNA or cells in the blood or other body fluids. Sensitivity can be improved by following certain strategies such as next-generation sequencing and increasing the yield of ctDNA extraction. Another important limitation is limited specificity; some genetic or epigenetic alterations detected in cfDNA may not be unique to neoplasm, thereby reducing the specificity for cancer diagnosis. This can, in turn, lead to increased false positivity [46]. Errors in sample collection techniques, handling, and processing can also affect the precision of liquid biopsy analysis. Currently, liquid biopsies, being a novel instrument in diagnosis, can be more expensive to perform when compared to traditional biopsies due to the need for specialized platforms and expertise for assay development and data analysis. As a result, the development of cost-effective methods and procedures is important for the widespread reach of liquid biopsy. Liquid biopsies lack quality control measures and standardized protocols. Regulatory hurdles remain for validating the utility of emerging liquid biopsy tests in clinical scenarios [47]. While liquid biopsy shows potential as a minimally invasive type of diagnostic tool, technical barriers include very low concentrations of cfDNA and exosomes in plasma. Considerable variability exists in sample collection methods and lack of standardization across isolation and detection platforms. Analytical sensitivity and specificity concerns persist, requiring rigorous validation in large cohorts with long-term follow-up [48]. The clinical utility must be demonstrated via trials and appropriate quality standards implemented to facilitate responsible translation of liquid biopsy-based tests. So, in summary, key challenges include technical factors like sensitivity and specificity, as well as standardization, tumor heterogeneity, cost, and regulatory issues. Overcoming these limitations will help translate liquid biopsies into routine clinical use.

Table 1 depicts the reference summary table.

**Table 1:** Summary table of the articles included in the review cfDNA: Cell-free DNA; TEPs: Tumor-educated blood platelets; ctDNA: Circulating tumor DNA; miRNA: MicroRNA; CTCs: Circulating tumor cells; EVs: Extracellular vesicles; ctRNA: Circulating tumor RNA; MRD: Minimal residual disease; ncRNAs: Non-coding RNAs; lncRNA: Long noncoding RNA; cMVs: Circulating microvesicles; CVDs: Cardiovascular diseases; AMI: Acute myocardial infarction; mNGS: Metagenomic next-generation sequencing; mcfDNA: Microbial cfDNA; TB: Tuberculosis; NGS: Next-generation sequencing

Sr No	Authors	Year	Interpretation
1.	Wan et al. [1]	2017	cfDNA and ctDNA are released into the bloodstream due to cell death and can be used for non-invasive genetic analysis and cancer testing.
2.	Collins et al. [2]	2015	The concept of precision medicine is not new, but recent advances in biotechnology have dramatically increased the prospects for broadly applying an individualized approach to disease prevention and treatment.
3.	Nikanjam et al. [3]	2022	ctDNA, cfDNA, and CTCs obtained through non-invasive biopsies enable personalized carcinoma management by detecting mutations, assessing tumor heterogeneity, predicting and assessing therapeutic response, finding MRD, and enabling faster detection of diseases
4.	Martins et al. [4]	2021	Liquid biopsies allow for the monitoring of cancer and for personalized, dynamic treatment approaches including diagnosis, prognosis, treatment selection, detecting minimal residual disease, predicting progression, and identifying resistance to reorient therapies.
5.	Siravegna et al. [5]	2017	Numerous components produced from tumors can be found in cancer patients' blood, such as exosomes, circulating tumor cells, and ctDNA. Liquid biopsy, which is the name for the collection and examination of circulating tumor components found in bodily fluids, allows for the minimally invasive tracking of cancer progression over time in a clinical setting.
6.	Pantel et al. [6]	2010	Ultrasensitive methods have enabled the identification of circulating tumor cells in the blood and bone marrow of patients suffering from cancer. Though invasive, bone marrow analyses and molecular profiling of these cells have provided insights into metastasis biology.
7.	Ma et al. [7]	2023	Liquid biopsy assesses tumor components like CTCs, cfDNA, ctDNA, ncRNAs, exosomes, and proteins in body fluids, thereby proving to be more advantageous over tissue biopsy including smaller sample size requirement, ability to repeatedly monitor molecular changes and tumor load in real-time, and inform personalized treatment adjustments.
8.	Praharaj et al. [8]	2018	CTCs are released from tumors and they act as seeds that can lead to metastatic lesions at other sites, as proposed in the seed and soil hypothesis by Stephen Paget in 1889.
9.	van de Stolpe et al. [9]	2011	CTCs are heterogeneous and differ between cancer types and within patients, with no single defining parameter. Combining parameters increases CTC detection specificity but decreases sensitivity.
10.	Krebs et al. [10]	2014	Due to intratumor heterogeneity, single-site biopsies might not be able to fully capture the genetic landscape of tumors. With the use of minimally invasive blood tests, CTCs provide a means of gaining insight into intratumor heterogeneity and cancer evolution. The growing use of targeted treatments emphasizes how crucial it is to check tumors for genetic abnormalities and comprehend how resistance develops throughout the course of the disease.
11.	Meng et al. [11]	2004	Every few hours, tumor cells must be replenished in the tissues to replace the CTCs that are dying. Therefore, in dormant candidates, there seems to be a balance between tumor multiplication and cell death for up to 22 years. We deduce that this is a possible mechanism for tumor dormancy.
12.	Ferreira et al. [12]	2016	Liquid biopsy including CTCs has the potential to significantly change the way cancer treatment is currently provided. Finding and isolating these uncommon cells using techniques that allow for downstream characterization and other uses is a major obstacle to maximizing the clinical potential of CTCs.
13.	Mandel et al. [13]	1948	The discovery of cfDNA fragments circulating in blood was first found by Mandel in 1948.
14.	Thierry et al. [14]	2014	In a study that took place in 1994, they analyzed cfDNA to detect certain genetic mutations and thereby established the concept of ctDNA.
15.	Chen et al. [15]	2021	ctDNA represents a subset of cfDNA that originates from apoptotic and necrotic primary tumor cells, metastatic lesions, or CTCs.
16.	Liao et al. [16]	2016	Cancer mutations found in the serum plasma are linked to vascular invasion and can be utilized in forecasting a shorter time for hepatocellular carcinoma patients to survive without recurrence. Tumor heterogeneity can be overcome by this type of biomarker. Furthermore, combining numerous mutations from various genes improves diagnostic performance.
17.	Vymetalkova et al. [17]	2018	It may be possible to reconstruct a tumor's genome from its ctDNA because it can act as a potential surrogate for the full genome. The genetic and epigenetic signatures found in ctDNA are consistent with those found in the original tumor and may provide insight into the epi-genetic spectrum unique to the tumor. This suggests that tumors directly release ctDNA.
18.	Melo et al. [18]	2015	Extracellular vesicles, also known as exosomes, are released by most cells and are composed of proteins and nucleic acids that are encapsulated in phospholipid bilayers. Tumor-derived material can be extracted from cancer patients' blood circulation using exosomes generated from cancer cells.
19.	Boeri et al. [19]	2011	After a spiral-CT screening experiment was completed and rigorously monitored, the expression profiles of miRNA in lung tumors, normal lung tissues, and plasma samples from patients were examined; miRNA expression patterns were found to significantly separate lung cancer, tumor histology and growth rate, and tumors from that of normal lung tissues.
20.	Best et al. [20]	2017	TEPs have shown promise as a non-invasive cancer detection biomarker source. Non-small-cell lung cancer could be accurately detected with TEP.
21.	Michela et al. [21]	2021	Highly sensitive genomic techniques can be used to evaluate the presence of circulating tumor proteins, ctDNA, and ctRNA.
22.	Salfer et al. [22]	2022	For several cancer types, urine circulating tumor DNA has demonstrated similar sensitivity when compared to blood or plasma cfDNA liquid biopsy in terms of detecting cancer mutations and other indicators. Therefore, urine cfDNA liquid biopsies might improve or replace tissue/blood biopsies in terms of informational value.
23.	Aro K et al. [23]	2017	Saliva contains biomarkers that make it well-suited for diagnosing a wide range of conditions such as oral cancers, pancreatic cancer, gastric cancer, and various systemic conditions.
24.	Patel et al. [24]	2022	Head and neck cancer (HNC) patients can undergo genomic and proteomic examinations by the identification, extraction, and analysis of metabolites, extracellular vesicles, CTCs, and ctDNA from their saliva.
25.	Aslebagh et al. [25]	2018	A new promising method for detecting breast cancer is monitoring of proteins and molecules in body fluids, such as saliva, breast milk, ductal fluid, tears, urine, and nipple aspirate. The only fluid that gives access to molecules from the surrounding breast environment as well as a significant number of exfoliated epithelial cells from the breast tissue is breast milk.
26.	Zhao et al. [26]	2018	The study demonstrated that synovial fluid-derived exosomal lncRNA PCGEM1 has the potential as a powerful indicator in differentiating early osteoarthritis from late-stage osteoarthritis
27.	Singh et al. [27]	2022	Point-of-care diagnostics are medical tests performed near patients to give fast results, enabling rapid clinical decisions. Key advantages include ease of use without specialized infrastructure, portability for bedside testing, and improved healthcare efficiency. Technologies like microfluidic biochips, paper strips, and portable spectroscopy devices are advancing point-of-care liquid biopsy to analyze disease markers from finger-prick blood.
28.	Cohen et al. [28]	2018	We used the CancerSEEK test on 1005 individuals who had clinically diagnosed non-metastatic malignancies of the breast, ovary, liver, stomach, pancreas, esophagus, colorectum, or lung. A median of 70% of the eight cancer types had positive results.
29.	Honoré et al. [29]	2021	MRD, or survival of cancerous cells following treatment with the goal of curing the disease, may be one factor in some patients' recurring illnesses. Standard radiological tests and clinical evaluations are not able to diagnose MRD. Tumor-specific blood alterations can be used to indirectly diagnose MRD. Thus, MRD may be detected by liquid biopsies, which may also enable the identification of tumor-specific microRNA, CTCs, or ctDNA.
30.	Rolfo et al. [30]	2021	Tracking genetic mutations in serial ctDNA measurements allows for the detection of emerging resistance, also enabling rapid adaptation of treatment plans.
31.	Reinert et al. [31]	2016	The current work provides additional evidence that suggests numerous possible clinical applications for tracking ctDNA levels during follow-up following colorectal cancer surgery.
32.	Hrebien et al. [32]	2019	Changes in ctDNA levels have predicted the prognosis and outcomes with different therapies for breast cancer.
33.	Abbosh C et al. [33]	2017	Serially taken ctDNA assessment enables early evaluation of chemotherapy, radiation, or surgical efficacy by monitoring molecular residual disease in lung cancer evolution
34.	Mannelli C et al. [34]	2019	The gold standard for diagnosing cancer nowadays is tissue biopsy, which is only practical when the lump is visible and yields consistent results. However, because liquid biopsy depends on the analysis of bodily fluids for operation, it is a potential experimental technology that has not yet been used in clinical practice but allows for early diagnosis. However, compared to tissue biopsy results, its results are less dependable since false positives and false negatives could happen.
35.	Badimon et al. [35]	2020	Phospholipid-rich blebs known as cMVs are secreted by most cells. There is increased release in response to cell activation and damage, representing the condition of the cell that produced them. Numerous research works propose the application of cMVs as prognostic markers for various stages and intensities of CVDs.
36.	Zhu et al. [36]	2023	The evaluation of cfDNA levels may be useful in diagnosing heart transplant rejection and predicting the severity of acute myocardial infarction.
37.	Devaux et al. [37]	2015	It has been confirmed that patients with AMI had higher levels of cardiomyocyte-enriched miR-208b and miR-499 when compared to those with other forms of acute chest pain.
38.	Blauwkamp et al. [38]	2019	We report the identification and quantification of microbial cfDNA in plasma from a large number of bacteria, fungi, viruses, and parasites using a next-generation sequencing technique that has been analytically and clinically validated. The test demonstrated more than 90% agreement with traditional culture methods and more frequently than all microbiological testing combined
39.	Han et al. [39]	2020	A growing number of infectious illness diagnostics are using mNGS of mcfDNA sequencing, which enables quick diagnosis, non-invasive sampling, and broad pathogen detection.
40.	Fernández-Carballo et al. [40]	2019	There is a promising use of cfDNA as a diagnostic tool which is non-invasive and can also be used in disease monitoring of TB.
41.	Zhou et al. [41]	2019	We identified several HIV-associated opportunistic infections using a unique NGS test on plasma samples. These findings were validated by traditional microbiological testing. Results from NGS tests were faster than those from traditional testing. This case series is the first to show how useful whole-genome NGS testing is for identifying opportunistic infections from plasma in HIV/AIDS patients.
42.	Perlin et al. [42]	2017	For a patient to recover from a serious fungal infection, the right antifungal medication must be used. Since there are so few antifungal medication classes on the market, patient management is severely hampered by drug resistance.
43.	Hong et al. [43]	2018	We study a non-invasive method for detecting fungal DNA in individuals who have been shown to have an invasive fungal infection on sequencing of cell-free plasma. When paired with clinical and radiographic data, this approach can help target anti-fungal medications without requiring tissue biopsy.
44.	Gaitsch et al. [44]	2022	Liquid biopsy has many applications in neurological illnesses, ranging from early diagnosis to treatment impact monitoring, relapse prediction, and outcome measurement in new therapy clinical trials. Liquid biopsy can also be used In order to better understand the aetiologies of complicated neurological illnesses like stroke and Alzheimer's disease and create more specialized treatment plans.
45.	Vaz et al. [45]	2022	It has been found that EVs from various neuronal cellular models can help understand key disease pathways, which in turn helps design novel therapeutic strategies. In the field of neuroscience, a number of researchers have employed exosome-like EVs that were separated from the neuronal models or other body fluids to investigate illness-related pathways or to find biomarkers for Parkinson's and Alzheimer's disease.
46.	Bronkhorst et al. [46]	2019	According to this study, the ctDNA analysis methods now in use, which mostly depend on the detection of DNA mutation, lack the necessary sensitivity and specificity for diagnosis.
47.	Armakolas et al. [47]	2023	To increase overall detection accuracy, it is essential to standardize procedures, increase top-quality antibodies along with high sample throughput, and thereby provide better detection resolution because the majority of organ-specific biomarkers are present in very small quantities and have not yet been identified.
48.	Jung et al. [48]	2010	As with tumor markers, the high aspirations as novel diagnostic tumor markers are limited by the low diagnostic sensitivity and specificity of cfDNA mutations. The primary issues that need to be addressed in future research include confirming the clinical validity of specific cfDNA mutations as possible cancer biomarkers for specific tumor types.

## Conclusions

Analysis of circulating biomarkers such as cfDNA, exosomes, and proteins through liquid biopsy techniques represents an emerging shift towards less invasive disease profiling in medicine. Liquid biopsies allow the detection and characterization of cancers, infections, cardiovascular diseases, neurological conditions, and other diseases through a simple, non-invasive blood sample. This enables applications including early diagnosis, serial monitoring of disease progression and treatment response, as well as identification of prognostic and therapeutic targets. By capturing tumor heterogeneity and evolution through repeat sampling, liquid biopsies can facilitate truly personalized precision medicine approaches tailored to each patient's molecular disease profile. While analytical and clinical validation remain ongoing challenges, liquid biopsy tests are rapidly transitioning from research into commercial products and clinical practice for a growing number of indications. With continued advances to improve performance combined with implementation of appropriate regulatory controls, liquid biopsy promises to transform disease management by reducing reliance on risky, expensive tissue biopsies. The realization of the full paradigm-shifting potential of liquid biopsies will rely on the demonstration of clinical utility across multiple disciplines of medicine beyond oncology.
